# Endogenous Cushing's Syndrome with Precocious Puberty in an 8-Year-Old Boy due to a Large Unilateral Adrenal Adenoma

**DOI:** 10.1155/2013/706989

**Published:** 2013-03-04

**Authors:** Muhammad Rajib Hossain, Md. Mashiul Alam, Junaid Nabi, Mahzabin Kibria

**Affiliations:** ^1^Department of Medicine, Shaheed Suhrawardy Medical College Hospital, Sher-e-Bangla Nagar, Dhaka 1207, Bangladesh; ^2^Department of Pediatrics, Dhaka Medical College Hospital, 100 Ramna, Dhaka 1000, Bangladesh; ^3^Department of Surgery, Shaheed Suhrawardy Medical College Hospital, Sher-e-Bangla Nagar, Dhaka 1207, Bangladesh

## Abstract

Adrenocortical tumors (ACTs) causing Cushing's syndrome are extremely rare in children and adolescents. Bilateral macronodular adrenocortical disease which is a component of the McCune-Albright syndrome is the most common cause of endogenous Cushing's syndrome. We report the case of a boy with Cushing's syndrome who presented with obesity and growth retardation. The child was hypertensive. The biochemical evaluation revealed that his serum cortisol levels were 25.80 *μ*g/dL, with a concomitant plasma ACTH level of 10.0 pg/mL and nonsuppressed serum cortisol on high-dose dexamethasone suppression test (HDDST) to be 20.38 *μ*g/dL. Computed tomography of the abdomen demonstrated a 8 × 6 × 5 cm left adrenal mass with internal calcifications. Following preoperative stabilization, laparotomy was carried out which revealed a lobulated left adrenal mass with intact capsule weighing 120 grams. Histopathological examination revealed a benign cortical neoplastic lesion, suggestive of adrenal adenoma; composed of large polygonal cells with centrally placed nuclei and prominent nucleoli without capsular and vascular invasion. On the seventh postoperative day, cortisol levels were within normal range indicating biochemical remission of Cushing's syndrome. On followup after three months, the patient showed significant clinical improvement and had lost moderate amount of weight and adrenal imaging was found to be normal.

## 1. Introduction

Adrenocortical tumors (ACTs) are quite rare in children and adolescents. Iatrogenic hypercortisolism is the most common cause of Cushing's syndrome (CS) in infancy and childhood [1]. In infants and children less than 7 years of age, adrenal tumors and predominantly malignant adrenal carcinoma constitute the most common causes of Cushing's syndrome [2] and that of those older than 7 years is ACTH secreting pituitary adenoma. ACTH-independent CS in children has been reported to be due to bilateral macronodular adrenocortical disease encountered in cases of McCune-Albright syndrome (MAS) [3]. The prevalence of the syndrome is reported to be between 1/100,000 and 1/1,000,000 and has been observed more commonly in females [4], with a tendency of severe presentation.

We report the case of an 8-year-old boy who was diagnosed with Cushing's syndrome due to a left sided adrenal adenoma who had presented with generalized obesity and growth retardation with features of precocious puberty. Subsequently, a left adrenal adrenalectomy was performed and clinical stabilization resulted in weight loss and biochemical resolution of Cushing's syndrome.

## 2. Case Report

**Figure 1:**
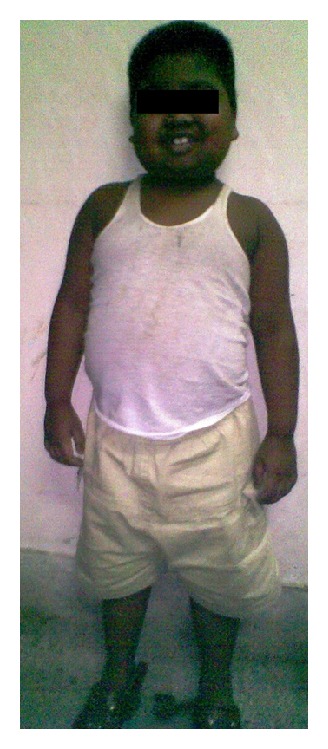
Typical appearance of Cushings syndrome (note moon face and abdominal distension).

**Figure 2:**
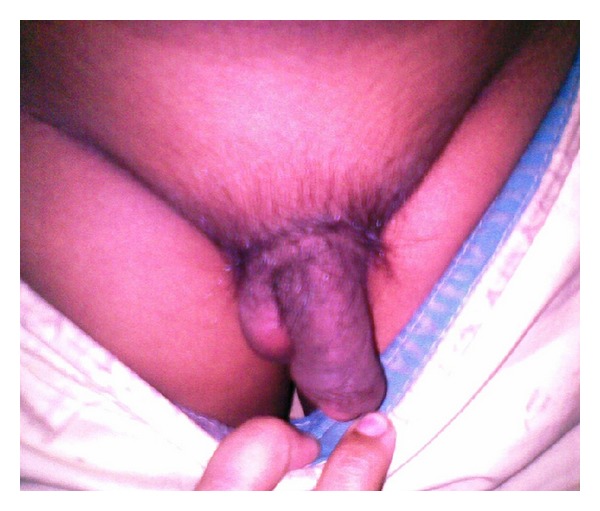
The unexpected finding of enlarged phallus more than normal for age and growth of child and coarse pubic hair.

An 8-year-old Bangladeshi boy presented to our out patient department (OPD) with complaints of gaining weight for the last 9 months. The parents were also concerned that the boy was not attaining the height according to his age. The child was born to nonconsanguineous parents at term, by normal vaginal delivery. Birth weight was 2.6 kg. Physical examination revealed a chubby boy with moon face and protruding abdomen (Figure 1). Increased body hair, striae, and other stigmata of MAS such as café-au-lait spots were absent. An unexpected finding was an abnormally large phallus with coarse pubic hair (Figure 2). On enquiry, the parents gave no history of steroid intake. His IQ scores were appropriate for his age and there was no history of voice change, and his sibling was in good health. The patient's body length was 92 cm (<3rd percentile; standard deviation score (SDS) −6.5) and his weight was 29 kg (80th percentile; SDS +1.6).

Blood pressure was 220/140 mmHg. Biochemical evaluation revealed a cortisol level of 25.80 *μ*g/dL with a concurrent plasma ACTH level of 10.0 pg/mL (within normal limits). His serum cortisol following high-dose dexamethasone suppression test (HDDST) (0.25 mg of dexamethasone every 6 hours for 48 hours (20 *μ*g/kg/dose)) was 20.38 *μ*g/dL and fasting blood sugar was 110 mg/dL (Table 1).

**Figure 3:**
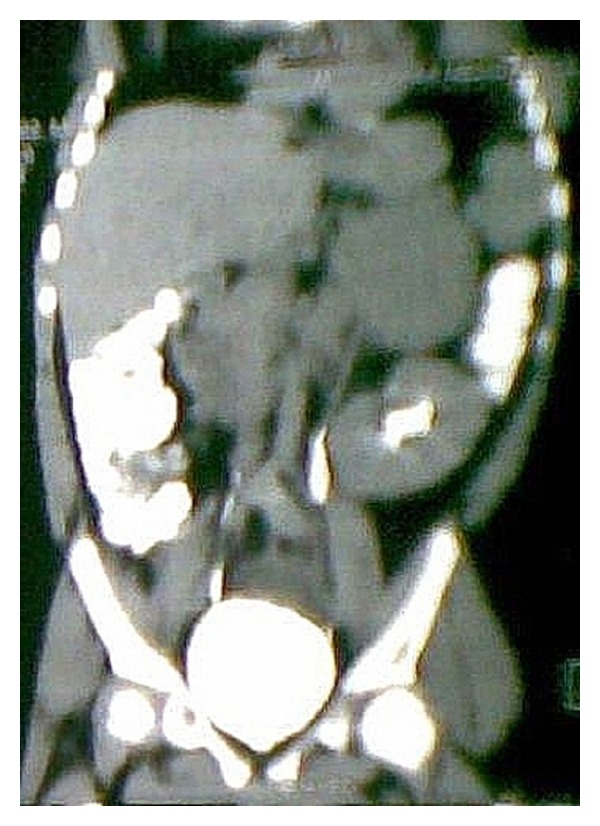
Contrast-enhanced abdominal computed tomography (CECT) scan in axial view revealed a large, well-circumscribed mildly enhancing adrenal mass (8 × 6 × 5 cm approx.) having few internal calcifications at left suprarenal region, which had displaced left kidney slightly downwards (arrow-mass; triangle-left kidney).

**Figure 4:**
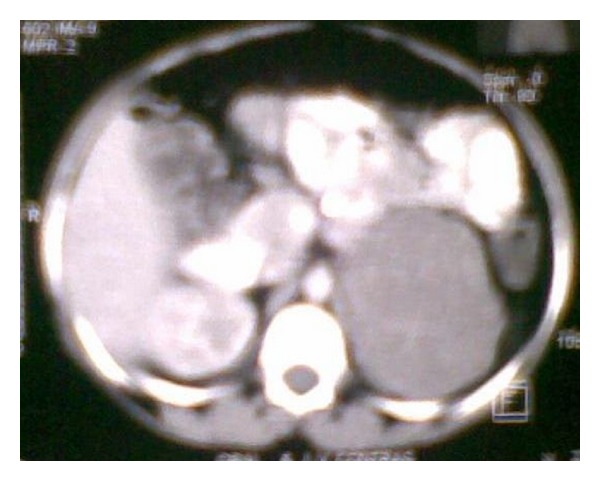
Enlarged view of CT scan image in cross section, showing a large well-circumscribed mildly enhancing mass having few calcification at left suprarenal region.

Ultrasonography revealed a left suprarenal mass with parenchymal disease of kidneys. Patient underwent a contrast-enhanced abdominal computed tomography (CECT) scan which divulged a large, well-circumscribed mildly enhancing adrenal mass (8 × 6 × 5 cm approx.) having few internal calcifications at left suprarenal region, which had displaced left kidney slightly downwards (Figures 3 and 4).

The patient underwent left adrenal adrenalectomy following laparotomy, which revealed a 8.2 × 6.3 × 5.2 cm lobulated mass with capsule being intact and weighing 130 gm. There were no adhesions to adjacent organs, no lymphadenopathy, and the inferior vena was spared. Histopathological evaluation revealed a benign cortical neoplastic lesion, suggestive of adrenal adenoma, composed of large polygonal cells with centrally placed nuclei and abundant pale eosinophilic cytoplasm. Some of the cells showed endocrine atypia albeit capsular and vascular invasion being absent.

In the postoperative management, the patient was given hydrocortisone (I/M) which was tapered off slowly. After one week, cortisol levels were within normal limits demonstrating biochemical remission of Cushing's syndrome. There was also moderate decline in the weight of the patient. The patient was discharge on post-operative day 10. Followup was done after three months and patient had lost more weight and adrenal imaging was normal.

## 3. Discussion

The most common cause of hypercortisolism with clinical manifestations of Cushing's syndrome is administrationof synthetic glucocorticoids [5]. Pediatric adrenocortical tumors (ACTs) are rare in infancy and occur primarily in children between one and five years of age (60%), with a peak in incidence below 4 years of age (0.4 cases per million). Nearly half of these ACTs are adrenocortical carcinoma [6]. Adrenal cortical tumors (ACTs) constitute less than 0.2% of all pediatric neoplasms and account for 6% of all adrenal tumors in children with an estimated incidence of 0.3 million population [7]. There seems to be a bimodal incidence of these tumors, with one peak at under 5 years of age and the second one in the fourth or fifth decades of life. Increased androgen production in infancy and early childhood ACTs is attributed to the structure of the adrenal gland at the time of birth when the inner fetal zone constitutes 85%–90% of the gland; the primary steroid product of the inner fetal zone is dehydroepiandrosterone sulphate [8].

The physical changes that occur in Cushing's syndrome such as the moon face, hirsutism, and acne as well as the bulging of the cervicodorsal region (buffalo hump) are a result of the intense action of the glucocorticoids which favor the accumulation of fat in the abdomen, chest, and face (central obesity). Growth hormone and *β*-adrenergic receptor antagonists also induce lipolysis which facilitates increase in triglycerides and free fatty acids. Hypertension is a consequence of increased renin substrate and sodium retention, facilitating the expansion of extracellular volume. In our case, the patient presented with precocious puberty which is in line with the observations made by Michalkiewicz et al. [9] who found in a registry of 254 pediatric patients with ACTs that 55% with virilization alone. Twenty-nine percent presented with mixed overproduction of adrenal hormones. Only 5.5% percent of this group presented with isolated Cushings syndrome, and this tended to occur in older children (median age 12.6 years).

Several laboratory investigations are helpful in establishing the diagnosis and differentiating between suprarenal or hypophyseal origin. These include serum cortisol levels, plasma ACTH, and high-dose dexamethasone suppression test (HDDST) which has better sensitivity [10]. Radiological evaluation includes ultrasonography, computed tomography (CT) scan of abdomen, and MRI of the brain. CT scan has been shown to be more sensitive in identification and localization of tumor mass [11]. The recommended procedure is surgical removal of the tumor (adrenalectomy) [12], which resulted in rapid weight loss in our patient. Post-operative hydrocortisone supplementation following surgery for adrenal adenoma causing CS is necessary as the contralateral adrenal gland is usually hypoplastic secondary to prolonged suppressed ACTH secretion from the pituitary due to CS.

Pediatric adrenocortical tumors (ACTs) are most commonly encountered in females and in children less than four years. But our case being an 8-year-old boy forms a rare presentation of endogenous Cushing's syndrome due to adrenal adenoma.

## Figures and Tables

**Table 1:** Biochemical evaluation of the patient.

Parameter	Value
An 8 am cortisol (*μ*g/dL) (4.4–22.6)	25.80
ACTH (pg/mL) (4–20)	10.0
HDDST Cortisol (*μ*g/dL) (41)	20.38
Fasting blood glucose (mg/dL) (119)	110
Sodium (mmol/L) (135–145)	138
Potassium (mmol/L) (3.0–5.5)	3.9
Creatinine (mg/dL) (0.4–1.2)	0.8
Hemoglobin (g/dL) (>11)	12.4

ACTH: adrenocorticotrophic hormone, HDDST: high-dose dexamethasone suppression test.
